# Facts and Fictions: Rethinking Vulnerability and Resistance in Late-Life Narratives and Why Age Matters

**DOI:** 10.1093/geroni/igaa057.3071

**Published:** 2020-12-16

**Authors:** Ulla Kriebernegg, Kate de Medeiros

**Affiliations:** 1 University of Graz, Graz, Steiermark, Austria; 2 Miami University, Oxford, Ohio, United States

## Abstract

Narrative gerontology examines the experience of aging through life stories and other first-person accounts. Literary gerontology explores cultural narratives (e.g., novels, films) that link us to our own aging through stories of others, real or imagined. Our paper focuses on narrative constructions of vulnerability, resistance, subjectivity and agency in life stories, interviews and fictional texts (e.g., Margaret Atwood’s short story “Torching the Dusties.”) It considers how aspects of vulnerability are embedded in stories and what they reveal about the cultural construction of age and aging or what makes us vulnerable as we age. Overall, our paper highlights the socio-cultural construction of vulnerability in narratives related to age and aging, focusing on the representation of vulnerability as a form of resistance and position of strength.

